# Circulating MAdCAM-1 and ITGB7 in Patients with Plaque Psoriasis and Eruptive Lichen Planus—Preliminary Data

**DOI:** 10.3390/biology10111129

**Published:** 2021-11-03

**Authors:** Anna Baran, Julia Nowowiejska, Tomasz W. Kamiński, Julita A. Krahel, Iwona Flisiak

**Affiliations:** 1 Department of Dermatology and Venereology, Medical University of Bialystok, Zurawia 14 St., 15-540 Bialystok, Poland; julia.nowowiejska@umb.edu.pl (J.N.); julita.leonczuk@gmail.com (J.A.K.); iwona.flisiak@umb.edu.pl (I.F.); 2 Pittsburgh Heart, Lung and Blood Vascular Medicine Institute, University of Pittsburgh, Pittsburgh, PA 15260, USA; kamins1@pitt.edu

**Keywords:** psoriasis, lichen planus, α_4_β_7_ integrin, MAdCAM-1, ITGB7, mucosal addressin cell adhesion molecule-1, integrin β_7_, lipids, liver, inflammatory bowel diseases

## Abstract

**Simple Summary:**

Psoriasis and lichen planus are common skin diseases which have similar clinical presentation and pathogenesis. Considering these dermatoses are frequent and decrease patients’ life quality, it is important to look for different markers indicating patients’ condition which can possibly affect the choice of the treatment. MAdCAM-1 and ITGB7 molecules and their serum levels in patients with psoriasis and lichen planus have never been studied before; therefore, we are the first trying to analyze it in order to develop the current state of knowledge on psoriasis and lichen planus to better help patients.

**Abstract:**

Plaque psoriasis (PSO) and lichen planus (LP) are skin diseases with some similarities in pathogenesis, comorbidities, and clinical presentation. Mucosal addressin cell adhesion molecule-1 (MAdCAM-1) and its ligand, α_4_β_7_ integrin, are involved in inflammatory bowel diseases and liver dysfunctions, which occur more frequently in PSO and LP. Serum MAdCAM-1 and ITGB7 levels in patients with plaque PSO and eruptive LP have never been studied before. The study included 42 patients with PSO, 13 with LP, and 23 controls. Serum molecules levels were evaluated using the immune–enzymatic method. ITGB7 concentration was not statistically different, both in patients with PSO and LP, compared to controls (both *p* > 0.05). MAdCAM-1 level was significantly lower in PSO subjects than in controls (*p* = 0.041), whereas in the LP group, a downward trend was observed (*p* = 0.088) with *p* = 0.0455 in ANOVA. Multiple linear regression revealed independent associations between ITGB7 and HDL and BMI and RBC in the LP group. In psoriatic patients with elevated CRP, there was an upward trend for MAdCAM-1, and also a positive correlation between MAdCAM-1 and WBC. ITGB7 and MAdCAM-1 cannot serve as markers of disease activity or liver pathology neither in patients with PSO nor LP. MAdCAM-1 might play a role as an inflammation indicator in PSO and a beneficial influence on the lipid profile in LP.

## 1. Introduction

Psoriasis (PSO) is a common skin disease which affects 3% of population on average [1]. Its etiology is still a subject of investigation, but genetic, autoimmune, autoinflammatory, and environmental factors are taken into account [2]. Flares of PSO may be induced by infection, stress, alcohol, or a particular drug’s intake [3,4,5]. Key disorders that can be observed in psoriatic skin are keratinocytes hyperproliferation, abnormal differentiation and apoptosis, along with neoangiogenesis [2]. There are many types of psoriasis described, with the most common being plaque PSO [1]. This type presents with erythematous–papular lesions with scaly surfaces, usually located on the extensor surface of elbows and knees, and also on the gluteal fold area [1]. PSO can also involve nails and scalp [1]. Nowadays, psoriasis is perceived not only as a skin disease, but also as a systemic disorder. Numerous research has proved its associations with a wide spectrum of comorbidities, namely metabolic syndrome (MS), liver diseases, arthritis, neoplasms, neurodegenerative disorders, nephropathy, or inflammatory bowel diseases (IBD) [1,6,7,8]. Our team had previously thoroughly investigated the relationship between PSO and different components of MS, looking for markers of metabolic complications [9,10]. We have also proved that psoriatic patients more often suffer from different sleep disorders [11]. Moreover, they have been discovered to be less efficient workers or pupils and their life quality is significantly worse than the general population [12]. Although there are various topical and systemic treatments available for psoriasis, it is often very hard to manage the condition and its multiorgan comorbidities [13,14]. Due to such broad physical comorbidity, along with mental disorders and impaired functioning in society, PSO has emerged to be a great medical, social, and economic burden.

Cutaneous lichen planus (LP) is a disease of uncertain etiology which occurs with a frequency of less than 1% of the population [15]. Several hypotheses are taken into account in LP pathogenesis. First, there are some histopathological and immunological similarities in LP and graft versus host disease (GVHD) [16]. LP also frequently appears after a severe stressful experience, infection, or particular drug’s intake [15]. Genetic susceptibility for LP is also considered [17]. There have been multiple variants of skin involvement in LP described, and besides skin, LP may affect mucosal membranes and nails. Cutaneous LP, which is the most common, manifests in its classic form as red-violaceous polygonal papules with so-called Wickham striae on their surface, which gives the impression of whitish opalescence. Lesions are usually limited to the extremities [15] and are often accompanied by pruritus [18]. One of other types is eruptive LP, which presents with numerous papules spread on the large body surface. Nowadays, LP is suspected to be associated with MS, similar to psoriasis [19].

PSO and LP have several similarities. First, they share a similar clinical picture because they both present with erythematous–papular lesions and may be accompanied by pruritus [18,20]. In both dermatoses, the Köbner phenomenon is observed [1]. Although the microscopic picture of these two dermatoses is different, sometimes some features may be similar (for instance, the presence of acanthosis) [3,15]. Their pathogenesis is not fully understood, but autoimmune factors are suspected as one of the possible paths, along with drugs, infections, and stress that can trigger the lesions’ occurrence [12,15]. Moreover, both PSO and LP have been related to components of MS [6,19].

Mucosal addressin cell adhesion molecule-1 (MAdCAM-1) is an adhesion molecule for lymphocytes which is expressed on mucosal venules, mainly in the intestines, stomach, esophagus, and pancreas, and less in the lungs, liver, and bladder [21]. It has also been found in the eyes, joints, and skin [22].

α_4_β_7_ integrin is a ligand for MAdCAM-1, and is expressed on CD4^+^ and CD8^+^ T lymphocytes, as well as on B lymphocytes, which allows them to be distinguished as unique gut-homing lymphocytes [21]. α_4_β_7_ integrin forms when integrin β_7_ (ITGB7) pairs with integrin α_4_ (ITGA4, CD49d) [23].

α_4_β_7_ integrin and MAdCAM-1 cooperate in lymphocytes’ recirculation between the blood vessels and the gastrointestinal tract [24]. MAdCAM-1, expressed on venules endothelium in tissues involved with inflammation, has been discovered to take part in leukocytes extravasation [25]. Research has shown that MAdCAM-1 is overexpressed in the sites involved with inflammatory processes in persons with Crohn’s disease (CD) and the interaction between α_4_β_7_ integrin and MAdCAM-1 is possibly even responsible for the state of chronic inflammation observed in intestinal diseases [21]. IBD has been proved to occur nearly twice as frequently in psoriatic patients than in the general population [26,27]. The integrity of the intestinal barrier in patients with psoriasis has also been investigated using I-FABP (intestinal fatty acid binding protein) and claudin-3. It revealed the dysfunction of the mentioned barrier and the influence of microbiota on the immune system and skin condition in psoriatic patients [28]. LP has also been described to coexist with IBD, especially ulcerative colitis [29]. Moreover, MAdCAM-1 has been discovered to be expressed in the liver in persons with diet-induced nonalcoholic steatohepatitis (NASH) and the loss of this molecule may even contribute to the decreased risk of NASH development. α_4_β_7_ integrin has been found to act inversely–its deficiency may lead to the increased risk of NASH development [23]. The frequency of NAFLD in psoriatic patients, which is closely related to MS, is estimated to be around 48–59% [27]. Both MAdCAM-1 and ITGB7 may also be assessed in their soluble form in human serum.

Plenty of similarities between PSO and LP, along with the immunological mechanisms involved in the pathogenesis of both diseases and the association between PSO, LP, and IBD or liver disorders, inspired us to explore the potential diagnostic role of ITGB7 and MAdCAM-1 in these interrelationships. To the best of our knowledge, this is the first study that evaluated serum MAdCAM-1 and ITGB7 levels in patients with PSO and LP. Our aim was to investigate serum proteins levels in these common papular dermatoses with regard to demographic or clinical data of the patients and laboratory indices of inflammation and metabolic disturbances. We intended to investigate these proteins as potential markers of inflammation, liver pathology, dyslipidemia, and disease severity (in PSO).

## 2. Materials and Methods

The overall study population included forty-two patients with active plaque-type PSO and thirteen with eruptive LP. Inclusion criteria were represented by a diagnosis made by a dermatologist of mild to severe PSO or LP. Exclusion criteria were as listed: age under 18 years, other types of psoriasis, pregnancy or breastfeeding, inflammatory or autoimmune disorders, and oncological or cardiometabolic diseases. None of the participants were under any dietary restriction. Washout periods for prior treatments were 3 months for systemic treatment and one month for topical therapy. In addition, twenty-three healthy volunteers matched by age, sex, and BMI were enrolled as controls. Peripheral blood samples were taken from all patients and healthy persons. The same dermatologist assessed the psoriasis area and the severity index (PASI) in all subjects with PSO. The psoriatic study group was divided according to severity of PSO into three subgroups: mild (PASI I) with PASI < 10 points included 13 patients, moderate (PASI II) with PASI 10–20 noted in 15 psoriatic patients, and severe form (PASI III) with PASI ≥ 20 achieved by 14 subjects. All participants were also divided into subgroups depending on BMI; group 0 were the controls, BMI 1 was related to normal-weight (BMI 18.5–24.9) and consisted of 15 psoriatic patients, group 2 was overweight (BMI 25–29.9), present in 15 subjects, and BMI 3 (BMI ≥ 30) meant obese patients, of which there were 12. Laboratory indicators of inflammation and metabolic disorders were assessed in all studied patients inter alia as C-reactive protein (CRP), white blood count (WBC), red blood count (RBC), platelets (PLT), serum fasting glucose, bilirubin, total cholesterol (Total Chol), high density lipoprotein (HDL), low density lipoprotein (LDL) and triglycerides (TG), and transaminases (alanine, ALT; asparagine, AST).

The experimental protocol obtained the approval of the local bioethical committee (Protocol number R-I-002/429/2017) and was performed according to the current version of the Helsinki Declaration, and each participant gave written informed consent before the onset of the study.

### 2.1. Serum Collection

Blood samples were collected from the study and control groups using Vacutainer tubes were left for 30 min to allow clotting before centrifugation for 15 min at 1000× *g*, after which the serum was separated and stored at −80 °C until use. MAdCAM-1 and ITGB7 levels were measured using the ELISA kit for MAdCAM-1 and ITGB7, Plasma MAdCAM-1 and ITGB7, Cloud Clone, SEB521Hu, and SEC100Hu, respectively. The minimum detectable dose of MAdCAM-1 was less than 0.33 ng/mL and of ITGB7, it was less than 0.055 ng/mL. The standard curve ranges were 0.78–50 ng/mL for MAdCAM-1, and for ITGB7, it was 0.156–10 ng/mL. Optical density was read at a wavelength of 450 nm. The concentrations were assessed by interpolation from calibration curves prepared with standard samples provided by the manufacturer.

### 2.2. Statistical Analysis

The normally distributed data were presented as mean ± 1SD, whereas the non-Gaussian data was the median (full-range). Normality of distribution was tested using the Shapiro–Wilk W test. The Student t test or nonparametric Mann–Whitney test were used to compare differences between CKD group and the control group. The χ2 test was used for categorical variables. The correlations were analyzed using Spearman’s rank correlation analysis or quasi-Newton and Rosenbrock’s regression analysis. Multiple regression analyses were performed using a stepwise model with a forward elimination procedure to determine the combined influence of variables on particular parameters of the measured system. Multiple regression analyses were performed based on previous results of Spearman’s rank correlation analysis or quasi-Newton and Rosenbrock’s regression analysis. A two-tailed *p* < 0.05 was considered statistically significant. Computations were performed using GraphPad Prism 6 (GraphPad Software; La Jolla, San Diego, CA, USA).

## 3. Results

The enrolled patients consisted of two groups: 42 persons with PSO: 31 males (73.8%) and 12 females (26.2%) of a median age of 48 (19–78) years old, and a mean PASI score of 7.67 ± 7.25. The other studied group were 13 subjects with LP: 4 males (30.8%) and 9 females (69.2%) of a median age 58 (21–77) years old. Baseline characteristics of the study population are presented in Table 1.

The mean value of BMI of LP patients was 30.16 ± 4.61, of PSO subjects: 28.11 ± 6.56 kg/m^2^, and of the controls: 25.45 ± 4.82 (Table 1). The mean basal PASI score for psoriatic patients was 7.67 ± 7.25 points.

Comparing the biochemical analysis between patients with LP and PSO, statistical differences were noted in LDL (low-density lipoprotein), serum level (*p* = 0.0008), and WBC (white blood cells) (*p* = 0.0112). Other parameters are listed in Table 2.

**Figure 1:**
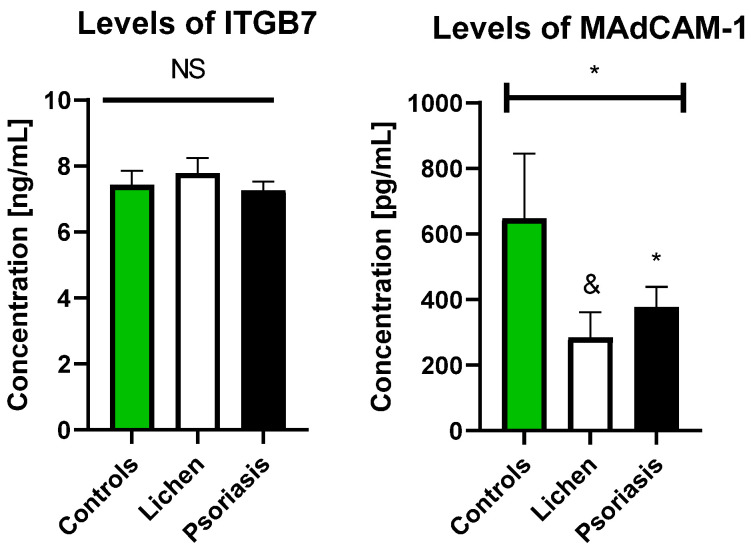
Levels of ITGB7 and MadCAM-1 in patients’ groups as compared to the control group. ANOVA for MAdCAM-1 (all three groups) *p* = 0.0455; * means controls vs. lichen = 0.088; & means controls vs. psoriasis = 0.041. NS, non-significant.

Serum ITGB7 concentration was not statistically different both in patients with PSO and LP as compared to the controls (both *p* > 0.05) (Figure 1). However, the MAdCAM-1 level was significantly lower in PSO subjects than in controls (*p* = 0.041), whereas in the LP group, a strong downward trend was observed (*p* = 0.088), along with *p* = 0.0455 achieved in an ANOVA test (Figure 1).

**Figure 2:**
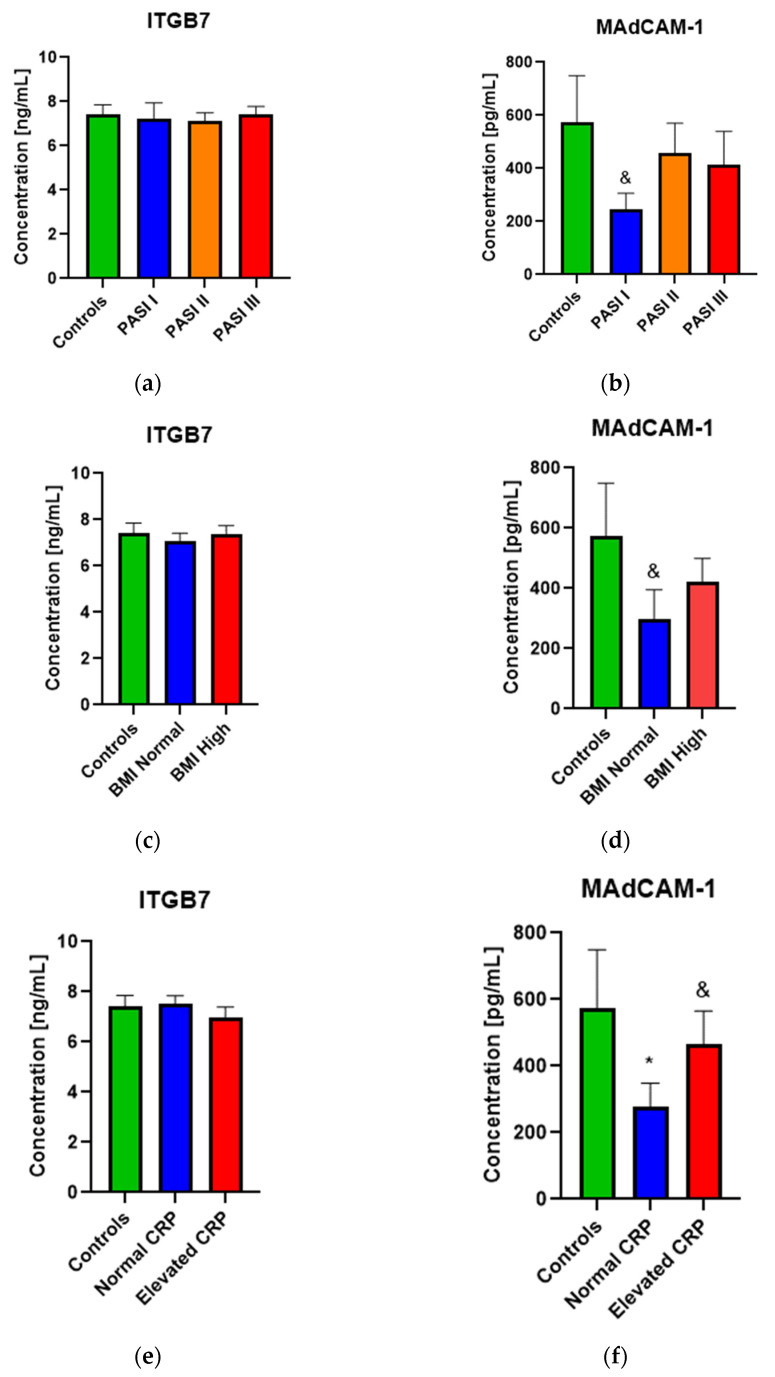
The levels of ITGB7 and MadCAM-1 in terms of PASI scoring, & means the existence of a trend between controls and PASI I subpopulation. *p* = 0.12 (**a**,**b**), BMI, & means the existence of a trend between controls and PASI I subpopulation. *p* = 0.11 (**c**,**d**), and CRP, * means *p* < 0.05 when comparing controls to normal CRP subgroup. *p* = 0.035, & means the existence of a trend between normal and elevated CRP subpopulation. *p* = 0.066 (**e**,**f**) allocations in psoriatic patients subgroups. PASI, psoriasis area and severity index; CRP, C-reactive protein; BMI, body mass index.

As for levels of the proteins evaluated in relation to the PASI subgroups, no statistical differences were found between both of them, except for the downward trend of MAdCAM-1 in patients with mild psoriasis (PASI I) as compared to the controls (*p* = 0.12) (Figure 2a,b). Similarly, no meaningful data between ITGB7 and MAdCAM-1 in accordance with BMI division were obtained; however, a downward trend of MAdCAM-1 level in psoriatic patients with normal weight (*p* = 0.11) as compared to the controls was noted (Figure 2c,d). Analyzing the relations of the evaluated proteins with CRP levels in PSO group, we found no statistical importance regarding ITGB7 (Figure 2e). However, the MAdCAM-1 level was significantly lower in psoriatic patients with a normal range of CRP than in the controls, whereas the assessed protein’s concentration was increased in psoriatic patients with elevated CRP as compared to the normal one (*p* = 0.066) (Figure 2f).

The assessment of relationships between the proteins and various laboratory parameters was performed and the most relevant data are listed in the Table 3.

Patients with LP ITGB7 tended to be negatively correlated with TG and RBC; however, this is without statistical significance (*p* = 0.08, *p* = 0.058, respectively) (Table 3a). In patients with PSO, we found a negative correlation between ITGB7 and age, and a positive one with RBC (*p* = 0.0011, *p* = 0.065, respectively). Furthermore, a negative correlation with AST activity was noted, however it was not statistical (*p* = 0.0623) (Table 3c). In the LP group, the MAdCAM-1 level insignificantly negatively correlated with TG, BMI, and PLT (respectively *p* = 0.088, *p* = 0.091, *p* = 0.096) (Table 3b). In PSO patients, MAdCAM-1 significantly positively correlated with WBC (*p* = 0.042) and positively with LDL or PLT, but without significance (respectively *p* = 0.056, *p* = 0.083) (Table 3d). The analysis with multiple linear regression revealed independent associations between ITGB7 levels and HDL, BMI, and RBC in patients with LP (Table 4).

No statistical correlations were found between the proteins studied and basic parameters in the controls (Table 5).

**Figure 3:**
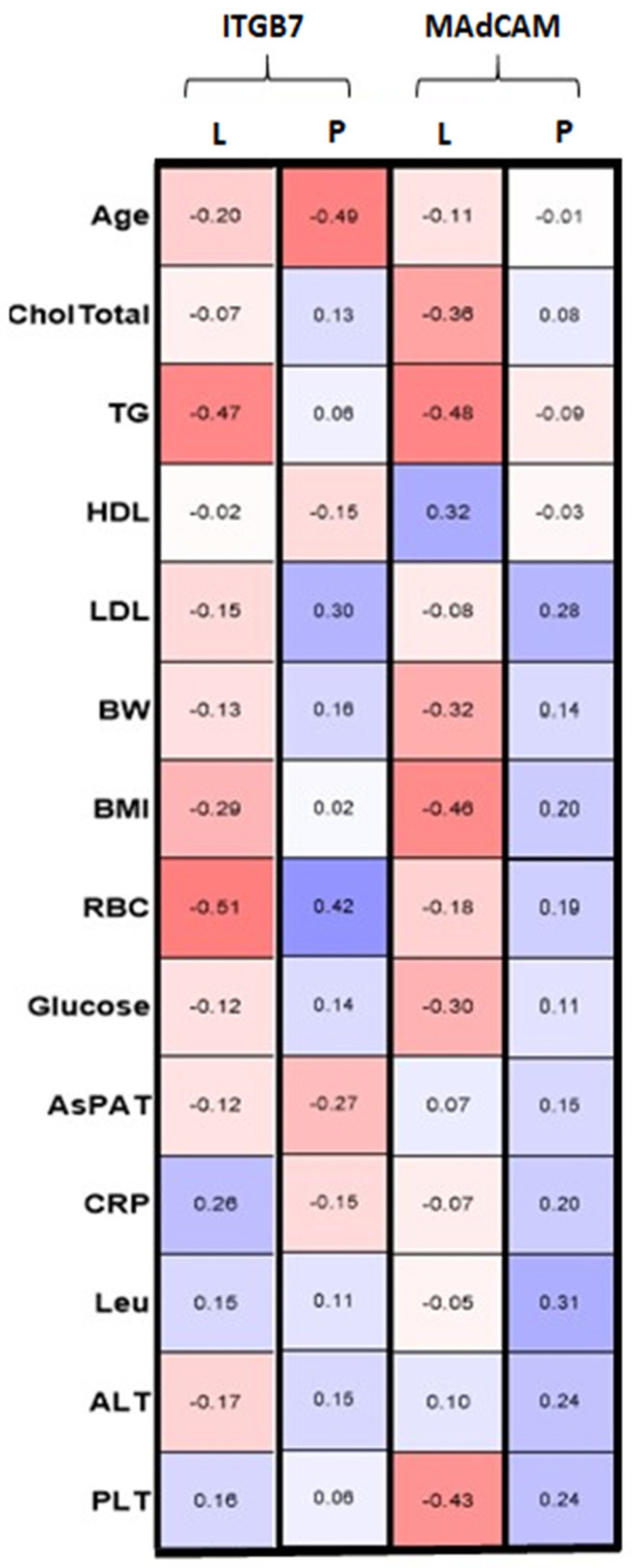
Correlations between ITGB7 and MAdCAM-1 concentrations and other parameters in patients with PSO (P) and LP (L). RBC, red blood cells; PLT, platelets; LEU, white blood cells; TG, triglycerides; HDL, high-density lipoprotein; LDL, low-density lipoprotein; CRP, C-reactive protein; ALT, alanine transaminase; AST, asparagine transaminase.

Interestingly, although the ITGB7 level does not differ between the studied groups and the controls, and MAdCAM-1 was significantly lower in PSO and with a downward trend in LP, both proteins positively correlated with each other in the controls and patients with LP (Table 6). Figure 3 shows correlations of the studied proteins with basic parameters in both groups of patients before and after treatment.

## 4. Discussion

To the best of our knowledge, neither MAdCAM, nor ITGB7 have been studied in PSO or LP so far. Vedolizumab, an antibody which targets the ITGB7 subunit and used as a drug in IBD has had some interesting results [30]. There are case reports of PSO flare after administration of this agent, although the exact mechanism is not understood [30,31]. It appears that somehow blocking off MAdCAM-ITGB7 interaction leads to PSO exacerbation, with possible unknown relations between the molecules and psoriasis pathogenesis. As far as we have searched, there are no reports regarding vedolizumab and LP.

Concentrations of soluble cell adhesion molecules (CAMs) have been proven to be elevated in patients with chronic inflammatory conditions, such as rheumatoid arthritis, systemic lupus erythematosus (SLE), neoplasms, bacterial and viral infections, as well as metabolic disorders such as atherosclerosis, hyperlipidemia, or diabetes mellitus [25]. Circulating CAM concentrations are considered as substitute markers of expression on the cell surface; therefore, they may serve as indicators of disease activity, progression, or response to treatment [25].

MAdCAM-1 has been proven to be secreted into human serum. Considering the nature of CAMs and the fact that MAdCAM-1 is also overexpressed in inflamed tissues, it could be assumed that its soluble form could serve as a marker of inflammation [32], taking into account elevated levels of CAMs in MS [33]. Moreover, one idea is that if skin may be one of the sites of extraintestinal manifestations of IBD, expression of MAdCAM-1 may attract cells and induce their state of inflammation [22]. Regarding our research, MAdCAM-1 serum concentration decreased significantly in PSO and with a downward trend in LP on the contrary, to the levels observed in patients with chronic inflammatory or metabolic conditions. However, considering no other studies on psoriatic patients exist, the results obtained cannot be disproved and require further in-depth research. MAdCAM-1 was also neither associated with PSO severity assessed with PASI nor with the MS laboratory markers (liver enzymes, lipid parameters, glycemia). As for inflammation indicators, there was no correlation between MAdCAM-1 and CRP observed in general, although in the group of psoriatic patients with elevated CRP, there was a trend for MAdCAM-1 concentration to be higher than in the group with normal CRP. There was also correlation between MAdCAM-1 and WBC noted. Apparently, this molecule cannot serve as a marker of disease activity or metabolic complications in patients with PSO, but it might play a role as an inflammation indicator in PSO patients; thus, it requires further investigations. In the group of LP patients, MAdCAM-1 seems to play a protective role on the metabolic status because it affects the lipid profile beneficially, but apparently this influence is still not sufficient considering the MAdCAM-1 concentration was significantly decreased in the general LP subpopulation. Therefore, there must be some other modifiers in that interplay. We want to highlight that concerdering/analyzing the absolute value of concentration of MAdCAM-1 in comparison to the controls, the concentration was twice as high. We have noted that in our controls, it was about twice as high, which could affect our observations.

The properties of various integrin’s subunits and CAMs have already been studied; some of them have been proven to be engaged in liver pathology [23]. Concentrations of soluble ITGB7 have not been studied in PSO or LP so far, but they have been investigated in children with atopic dermatitis (AD). It was shown that in such patients’ serum, ITGB7 levels were elevated and can become markers of AD activity [34]. In our investigation, ITGB7 concentration was not significantly different between psoriatic patients with diverse skin lesion severity in PASI; therefore, it cannot serve as a marker of PSO severity. The analysis of ITGB7 suggests that its serum concentration in patients both with PSO and LP cannot serve as a marker of inflammation in these diseases. It is the more probable considering there was no difference in ITGB7 concentrations between groups of normal and elevated CRP in psoriatic patients. ITGB7 serum levels also seem to not be associated with BMI in psoriatic patients. It also appears that the concentration of ITGB7 in psoriatic patients is higher in younger patients and decreases with aging. We have noticed the negative correlation between ITGB7 serum concentration and age; therefore, older patients may tend to have lower concentration of such ITGB7. It has to also be taken into account that we have investigated serum ITGB7, which is only one part of α_4_β_7_ integrin, the right ligand for MAdCAM-1, so there might be some aberrations compared to the whole α_4_β_7_ integrin’s properties.

Previous research performed in different inflammatory diseases have shown that the inhibition of the interaction between MAdCAM and ITGB7 leads to a reduction in immune response processes [23]. Although MAdCAM-1 and ITGB7 have been described to be involved in the NAFLD and NASH development, which are closely related to psoriasis, we found no correlation between these two molecules and markers of liver function (transaminases activity). Therefore, we might conclude that they cannot serve as predictors of liver pathology in patients with PSO and LP. Further, larger studies are needed in order to confirm these results or look for other modifiers of these relations.

We found three parameters independently associated with ITGB7 in LP patients: BMI, RBC, and HDL concentration. Considering low ITGB7 may predispose to NASH development, it matches low HDL concentrations and low RBC. The number of erythrocytes, which transport hemoglobin and are involved in oxygenation homeostasis, may decrease under the conditions of inflammation and oxidative stress [35]. In the case of NASH/NAFLD development, when ROS are prominent and lead to cell death and tissue injury, they may damage erythrocytes as well, which corresponds with low ITGB7 concentrations [36]. Similarly, low HDL, as a component of MS, may be associated with other metabolic disorders, such as NASH/NALFD. Apparently, in patients with LP, low RBC and HDL concentration may indirectly indicate low ITGB7 and increase the possibility of liver disorders.

The data on soluble MAdCAM-1 and ITGB7 is scarce; more so in PSO or LP, in which have never been studied before, so we have little research for comparison. Moreover, none of our patients suffered from IBD, so the molecules we investigated are a reflection of changes present only in PSO and LP, not affected by IBD for which MAdCAM–ITGB7 interactions have been probably most prominently described so far. Perhaps, despite some similarities between these dermatoses and IBD, soluble MAdCAM-1 and ITGB7 do not act by analogy to what we can expect in IBD or in NAFLD. To back up our findings, we have found a similar observation in one study conducted by Vavricka et al. who assessed, among others, MAdCAM-1 expression in IBD and PSO. They reported MAdCAM-1 overexpression in IBD but not in inflammatory skin diseases [37]. These authors advocate that only isolated inflammation in the intestines leads to MAdCAM-1 overexpression, not inflammatory conditions that involve skin [37].

As for other our limitations, it was a single-center study with a relatively small number of patients. Our research was based on the assessment of soluble MAdCAM-1 and ITGB7 instead of their expression in tissues. Moreover, we have investigated only ITGB7 instead of the whole α_4_β_7_ integrin as a ligand for MAdCAM-1. We have only assessed laboratory parameters and have not performed imaging tests (e.g., liver) in our subjects. Our outcomes remain preliminary and further investigations are needed to obtain more in-depth information.

In the future, we would like to extend our research in different ways. First, we would like to assess not only serum concentration, but also tissue expression of MAdCAM-1 and ITGB7. Moreover, we would like to perform imaging on the liver and look for associations between these findings and the above investigated molecules’ concentrations. Lastly, we would try to introduce some dietary interventions in patients with PSO and LP to see how they influence MAdCAM-1 and ITGB7 concentrations, along with evaluating the relations within various systemic antipsoriatic therapies.

## 5. Conclusions

ITGB7 cannot serve as a marker of disease activity, inflammatory processes, or liver pathology neither in patients with PSO nor LP. MAdCAM-1 cannot serve as a marker of disease activity, dyslipidemia, or liver pathology in PSO, but it might play a role as an inflammation indicator. In patients with LP, MAdCAM-1 seems to play a protective role on the lipid profile, but there must be other modifiers in that interplay, which requires further research.

## Figures and Tables

**Table 1:** Baseline characteristics of patients and controls.

Parameter/Group	Controls (*n* = 23)	LP (*n* = 13)	PSO (*n* = 42)
Age	**30 (25–65)**	58 (21–77) *	48 (19–78) *
Sex (M/F)	12/11 *	4/9 *	31/12 *
Body weight	77.78 ± 16.89	83.77 ± 10.57	84.83 ± 21.05
Height	174.7 ± 9.1	167 ± 4.4	173.7 ± 9.53
BMI	25.45 ± 4.82	30.16 ± 4.61	28.11 ± 6.56

Age: *p* = 0.0271; sex: *p* = 0.0203; BMI: *p* = 0.0567; BMI = body mass index. **Bold font** means the existence of the statistical significance. * means the existence of a statistically significant difference with *p* < 0.05.

**Table 2:** Biochemical analysis of the studied groups.

Parameter/Group	LP (n = 13)	PSO (n = 42)
Total chol	185.9 ± 38.69	172.1 ± 26.85
TG	133.8 ± 31.48	137.3 ± 50.79
HDL	50.71 ± 12.22	48.79 ± 15.39
LDL	133.8 ± 18.96 ***	102.7 ± 26.88 ***
RBC	4.61 ± 0.44	4.52 ± 0.56
WBC	5.91 ± 1.74 *	7.94 ± 2.61 *
PLT	225.9 ± 51.24	247.2 ± 73.8
Glucose	93 (76–136)	84 (12–152)
AST	19.15 ± 5.01	30.4 ± 19.8
ALT	20 (11–42)	21 (5–153)
CRP	1.97 (1–7.11)	3.12 (1–56.29)
PASI	-	7.67 ± 7.25

*/*** means the existence of a statistically significant difference between the groups with *p* = 0.0008 for LDL, and for WBC: *p* = 0.0112; RBC, red blood cells; PLT, platelets; WBC, white blood cells; TG, triglycerides; HDL, high-density lipoprotein; LDL, low-density lipoprotein; CRP, C-reactive protein; ALT, alanine transaminase; AST, asparagine transaminase, Total chol, total cholesterol; and PASI, psoriasis area and severity index. **Bold font** means the existence of the statistical significance.

**Table 3:** Correlations between ITGB7 (a,c), MadCAM-1 (b,d), and patients’ parameters in LP and PSO groups. (R/*p*-value).

Parameter	R	*p* Value
(a) LP—ITGB7		
TG	−0.478	0.088
RBC	−0.505	0.058
(b) LP—MAdCAM-1		
TG	−0.478	0.088
BMI	−0.456	0.091
PLT	−0.426	0.096
(c) PSO—ITGB7		
Age	**−0.492**	**0.001**
RBC	**0.418**	**0.006**
AST	−0.322	0.062
(d) PSO—MAdCAM-1		
LDL	0.284	0.056
PLT	0.244	0.083
WBC	**0.315**	**0.042**

**Bold font** means the existence of a statistically significant difference, *p* < 0.05. RBC, red blood cells; PLT, platelets; WBC, white blood cells; TG, triglycerides; HDL, high-density lipoprotein; LDL, low-density lipoprotein; AST, asparagine transaminase.

**Table 4:** Multiple linear regression independent association between ITGB7 levels and included variables in patients with lichen planus.

Variable	|t|	*p* Value	*p* Value Summary
Total Chol	0.2018	0.8451	NS
TG	0.9389	0.3753	NS
HDL	**3.167**	**0.0132**	*
LDL	1.323	0.2224	NS
PASI	1.569	0.1553	NS
WBC	0.3854	0.7100	NS
PLT	0.6354	0.5429	NS
BMI	**4.567**	**0.0018**	**
RBC	**3.643**	**0.0066**	**
Glucose	1.372	0.2072	NS
AST	1.247	0.2476	NS
CRP	0.3216	0.7560	NS

*/** means *p* < 0.05/*p* < 0.01, respectively. NS, non-significant. RBC, red blood cells; PLT, platelets; WBC, white blood cells; TG, triglycerides; HDL, high-density lipoprotein; LDL, low-density lipoprotein; CRP, C-reactive protein; ALT, alanine transaminase; AST, asparagine transaminase, Total chol, total cholesterol; PASI, psoriasis area and severity index. **Bold font** means the existence of the statistical significance.

**Table 5:** Correlations between ITGB7 and MadCAM-1 with basic parameters in controls.

	Age	Body Weight	Height	BMI
ITGB7	0.389/*p* = 0.098	−0.281 NS	−0.154 NS	−0.235 NS
MadCAM-1	−0.466/*p* = 0.12	0.238 NS	0.117 NS	0.187 NS

NS, non-significant.

**Table 6:** Correlations between both proteins in studied groups.

ITGB7 vs. MadCAM-1	Controls	LP	PSO
R/*p* value	0.699/*p* = 0.002 **	0.61/*p* = 0.03 *	0.274/*p* = 0.083

*/** means the existence of a statistically significant difference. **Bold font** means the existence of the statistical significance.

## Data Availability

Data available on the request from the authors.
